# Effect of Ozone Treatment on the Quality of Sea Buckthorn (*Hippophae rhamnoides* L.)

**DOI:** 10.3390/plants10050847

**Published:** 2021-04-22

**Authors:** Anita Zapałowska, Natalia Matłok, Miłosz Zardzewiały, Tomasz Piechowiak, Maciej Balawejder

**Affiliations:** 1 Department of Land Management and Environmental Protection, University of Rzeszów, St. Ćwiklińskiej 1a, 35-601 Rzeszów, Poland; 2 Department of Food and Agriculture Production Engineering, University of Rzeszow, St. Zelwerowicza 4, 35-601 Rzeszów, Poland; nmatlok@ur.edu.pl (N.M.); mzardzewialy@ur.edu.pl (M.Z.); 3 Department of Chemistry and Food Toxicology, University of Rzeszow, St. Ćwiklińskiej 1a, 35-601 Rzeszów, Poland; tom_piech@interia.pl (T.P.); maciejb@ur.edu.pl (M.B.)

**Keywords:** ozone, sea buckthorn, mechanical properties, microbial load, bioactive components

## Abstract

The aim of this research was to show the effect of the ozonation process on the quality of sea buckthorn (*Hippophae rhamnoides* L.). The quality of the ozonated berries of sea buckthorn was assessed. Prior to and after the ozone treatment, a number of parameters, including the mechanical properties, moisture content, microbial load, content of bioactive compounds, and composition of volatile compounds, were determined. The influence of the ozonation process on the composition of volatile compounds and mechanical properties was demonstrated. The ozonation had negligible impact on the weight and moisture of the samples immediately following the treatment. Significant differences in water content were recorded after 7 days of storage. It was shown that the highest dose of ozone (concentration and process time) amounting to 100 ppm for 30 min significantly reduced the water loss. The microbiological analyses showed the effect of ozone on the total count of aerobic bacteria, yeast, and mold. The applied process conditions resulted in the reduction of the number of aerobic bacteria colonies by 3 log cfu g^−1^ compared to the control (non-ozonated) sample, whereas the number of yeast and mold colonies decreased by 1 log cfu g^−1^ after the application of 100 ppm ozone gas for 30 min. As a consequence, ozone treatment enhanced the plant quality and extended plant’s storage life.

## 1. Introduction

The sea buckthorn (*Hippophae rhamnoides* L.) is a spiny, deciduous shrub which belongs to the oleaster family (*Elaeagnaceae Juss*.) The fruits are rich in a variety of phytochemicals with physiological properties, such as lipids, carotenoids, ascorbic acid, tocopherols, and flavonoids [1,2]. A characteristic feature of *Hippophae rhamnoides* L. is intense orange berries that densely overgrow the shoots. A peculiar smell is caused by the presence of chemical compounds such as alcohols, aldehydes, ketones, and terpenes [3]. Sea buckthorn contains carotenoids, flavonoids, phospholipids, tannins, vitamins, and macro- and microelements [4]. Due to its abundance of the biologically active compounds, the sea buckthorn is widely used as a human health-promoter. This plant is a valuable material, particularly in pharmaceutical, cosmetic, and food industries [5]. Ozone, a highly reactive triatomic form of oxygen, is a powerful agent able to degrade organic substances. Gaseous ozone, due to its properties, has been approved by the Food and Drug Administration for direct contact with food, and can have a significant impact on the quality of the fruit. Numerous studies have confirmed its beneficial effect on the quality of products, evidenced in the extension of their shelf life [6,7]. Due to ozonation, select parameters of plant materials, such as antioxidant potential, polyphenol content, or enzymatic activity, are modified. Many studies have confirmed that ozone treatment inhibits the growth of microorganisms responsible for fruit spoilage, and reduces the loss of nutritional and sensory value of fruit during storage [8,9,10,11,12,13,14,15,16,17]. These studies have shown that the use of ozonated water is only effective in combination with blanching [8,13]. Using ozone gas is more effective, but it is necessary to change the atmosphere with other gases [9,12]. A plant composition change may occur as a result of the ozonation process [14,15]. This gas, as confirmed by numerous studies undertaken by the Environmental Protection Agency, is considerably safer than chlorine or other sanitizers [18]. In contact with solid materials, it decomposes into molecular oxygen and does not cause additional fouling.

The studies undertaken to date on the utilization of ozone treatment in fruit storage have principally concerned the effect of ozonation on the level of microbial contamination, biological activity of the fruit, and fruits’ sensory characteristics [14,15,19]. However, the available data on changes in the bioactive compounds of ozonated fruits are variable and difficult to compare because they are related to different factors, forms, and concentrations of ozone, in addition to different fruits and environmental conditions in which they are produced [20].

No reports have been published about the effect of ozone treatment on the quality of sea buckthorn (*Hippophae rhamnoides* L.). To obtain factual information, we conducted a survey, the results of which are shown below. The aim of the research was to show the effect of the ozonation process on the quality of sea buckthorn (*Hippophae rhamnoides* L.).

## 2. Results and Discussion

### 2.1. Moisture Content

Sea buckthorn berries consist of more than 80% water, which fluctuates greatly during storage. The moisture content depends largely on the storage conditions. Fruit stored in the same conditions should therefore maintain similar quality and strength parameters, which are dependent on the moisture content. The fruit shelf life can be influenced by the metabolic system being activated. This can be performed by adjusting the storage to proper conditions (appropriate storage temperature) or by making use of both biotic and abiotic factors (in the form of nutrition, cutting, light, water, etc.). One such factor (abiotic) is the ozone gaseous form, which may be used to slow the ripening process. The findings of our experiment show the effects on the ozone concentration and its duration on the moisture content.

**Figure 1:**
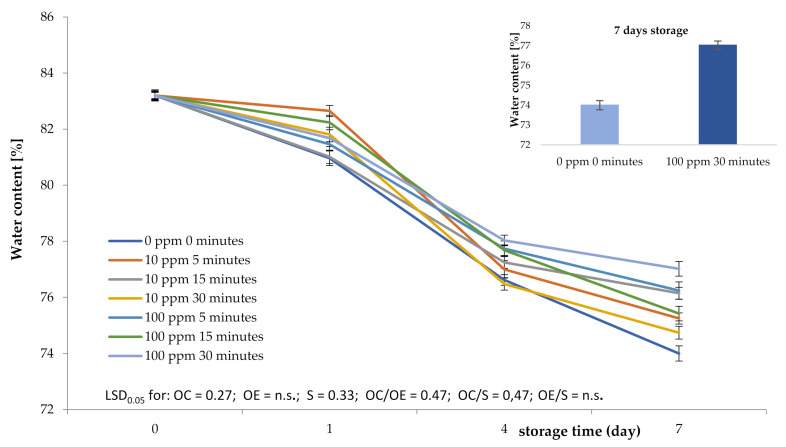
Effect of the ozone treatment concentration (OC), ozone exposure time (OE), and storage time (S) on moisture content of *Hippophae rhamnoides* L. fruit.

The applied ozone treatment had a direct influence on the sea buckthorn shelf life. The observed changes in moisture content depended on ozone concentrations and exposure time (Figure 1). The largest water loss, which amounted to 5.20%, was observed in the control sample (0 ppm, 0 min) on the 7th day of storage, and the lowest water loss of 3.65% was recorded in the samples subjected to ozone treatment at a rate of 100 ppm, for the duration of 30 min. The performed statistical analysis showed a significant influence of ozone concentration and ozone exposure time (LSD_0.05_ (least significant difference) 0.47) on the moisture content of the sea buckhorn berries. This corresponds to the observation of the lowest microbial load for fruit subjected to ozonation under these conditions. It is most likely that this was caused by a decrease in the activity of microorganisms on the surface of this fruit. Ozone, as an agent that disinfects fruit surfaces, affects the activity of microorganisms and the storage process. Microbial inactivation by ozone, mainly due to the rupture of its cellular membranes, prevents damage of the fruit surface, leading to a reduction in water loss and increasing the membranes’ mechanical strength. The influence of the ozonation process on the reduction of water loss in stored plant material has been also confirmed in other studies. Zardzewiały et al. [21] observed a similar effect of ozone treatment applied to rhubarb petioles. The authors recorded the lowest water loss for the exposure of 30 min with an ozone concentration of 100 ppm. The influence of the ozonation process on improving quality parameters and reducing water loss in stored ground cucumbers was also confirmed by Gorzelany et al. [22]. Antos et al. [23], using ozone on stored apples, observed a similar relationship.

### 2.2. The Mechanical Properties

The mechanical parameters determine the quality of the raw material plants, which is important in terms of the storage ability. During the storage time, all ozonated sea buckthorn berries showed a higher resistance to the destructive force (Fmax) compared to non-ozonated fruits (Table 1). The mean values of indentation force in the control sample amounted to 6.84 N. To correlate moisture content with selected mechanical properties, these parameters were measured at the same time of storage (on the 1st, 4th, and 7th days). The results of the test show that water loss from cellular structures directly affects the parameters of the mechanical properties. The value of destructive force (Fmax) subjected to these conditions after the 1st day of storage was 10.1% higher compared to non-ozonated fruits. Significantly better results were recorded on the 4th and the 7th day of storage, when the value of Fmax was 36.3% and 58.6% higher, respectively, compared to the control sample (non-ozonated). The result shows that, as in the case of moisture content, the highest measured parameter was recorded on the 7th day following treatment at the ozone rate of 100 ppm for 30 min (Fmax = 9.69 N). This was likely the result of less damage to the ozonated surface of the fruit caused by microbes. Increasing the resistance of ozonated sea buckthorn berries to the destructive force has been associated with the alteration of the fruit surface due to ozone gas. Similar relationships were observed on the recorded deformation of sea buckthorn berries to the point of destruction (dl to Fmax) and destructive force (Fmax/Lmax). These parameters were significantly higher in the case of fruit exposed to gaseous ozone. The performed statistical analysis showed a significant influence of ozone treatment concentration, ozone exposure time, and storage time on the mechanical parameters (Table 1). The impact of the ozonation process on selected mechanical parameters of the plant material was also confirmed in other studies. Zardzewiały et al. [21] observed a similar effect of the ozonation process on the mechanical resistance of rhubarb in the compression test. The best results were found in the case of the material subjected to the process of ozone treatment for 15 min with a concentration of 10 ppm. Gorzelany et al. [22], using gaseous ozone for post-harvest ground cucumber, obtained a material with improved mechanical and sensory properties compared to the control. The increase in the parameters of the destructive power of ozonated apples was noted by Antos et al. [23]. Using cyclic gaseous ozone on apples, the authors observed a similar relationship between the conditions of the ozonation process and the mechanical parameters of the stored fruit. 

### 2.3. Content of Bioactive Compounds

**Figure 2:**
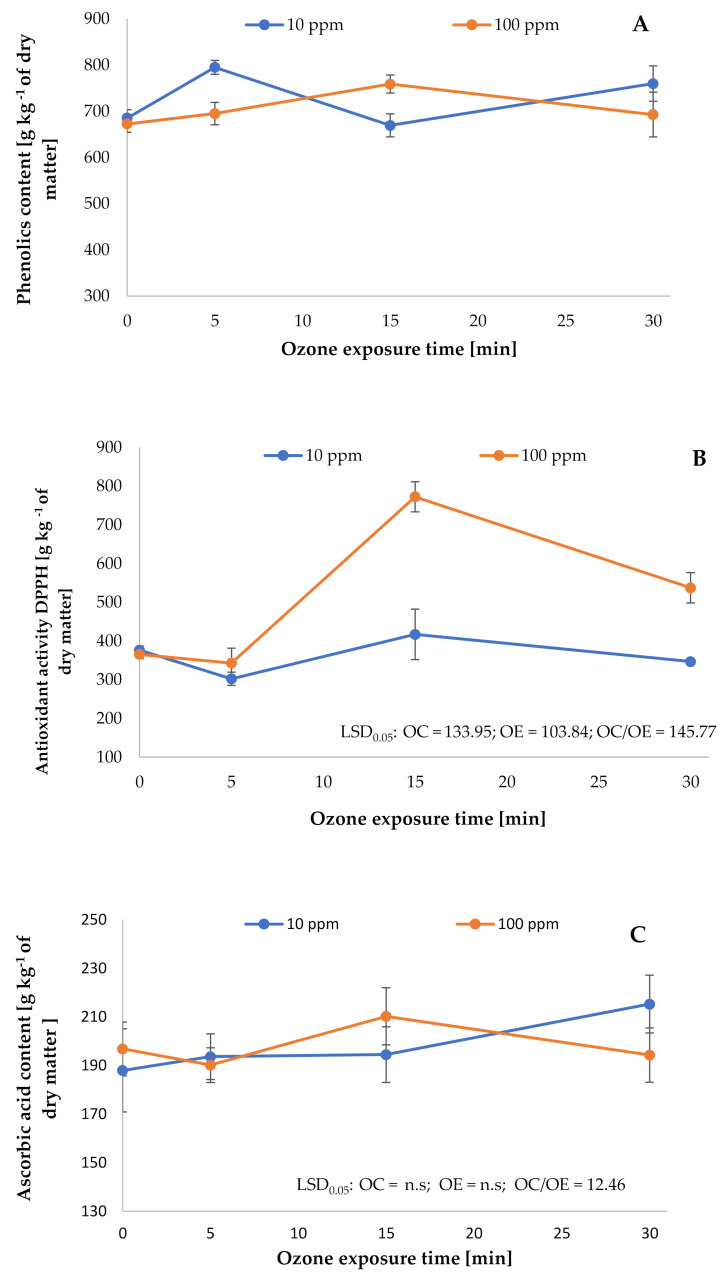
The impact of ozonation process on the polyphenolic content (**A**), antioxidant activity against DPPH radical (**B**), and total ascorbic acid content (**C**) in *Hippophae rhamnoides* L. fruit (*n* = 3). Note: LSD for α = 0.05 for the impact of ozone treatment concentration (OC), ozone exposure time (OE), and the interaction between tested parameters (OC)/(OE).

The results of the present study show increased total polyphenol content after 24 h (Figure 2A). This was determined by the duration of the process and the ozone concentration. The best results were observed when the plant was exposed to ozone for 5 min with an ozone concentration of 10 ppm. In this case, the change in the total polyphenol content was 15.9% higher compared to the control sample (non-ozonated fruit). When higher ozone concentrations of 100 ppm were used, the best results were achieved after 15 min. In this case, an increase in total polyphenol growth of more than 12.8% was found in the ozonated plant. A strong relationship with ozone dose was observed in the case of antioxidant potential (Figure 2B). The antioxidant capacity of the fruit 24 h after the ozone treatment of 100 ppm for 15 min was higher by 11.5% compared to the control sample (non-ozonated fruit). A quantity of 100 ppm of ozone significantly disturbed the fruit balance, which caused oscillation of the content of components, particularly polyphenols. Such oscillations were not observed for the antioxidant potential. Statistical analysis showed the impact of the ozone concentration and ozone exposure time on the total content of polyphenols in sea buckthorn berries (LSD_0.05_ = 53.07), and their antioxidant properties (LSD_0.05_ = 145.77). In addition, the antioxidant potential depended significantly on the time of the ozonation (LSD_0.05_ = 103.84) and ozone concentrations (LSD_0.05_ = 133.95). The increase in the concentration of secondary metabolites in ozonated sea buckthorn berries after 24 h of storage can be attributed to metabolic changes due to the occurrence of oxidative stress caused by ozone. The effect of ozone on the total polyphenol content and antioxidant potential of the ozonated fruit has been confirmed by other scientists. Rodoni et al. [24] recorded a 50% increase in the total polyphenol content compared to the non-ozonated fruit, on the sixth day after the ozone treatment, while examining the effect of 10 min ozone exposure at a dose of 10 μL L^−1^ on tomato fruits. The increase in total polyphenol content was also observed in the case of the ozonated fruits of *Carica papaya* L. [25]. Alothman et al. [26], using ozone at a flow of 8 ± 0.2 mL s^−1^ for 0, 10, 20 and 30 min to ozonate pineapple and banana fruits, found an increase in the total polyphenols in ozonated fruit. The best results were achieved when the ozonation process was conducted for 30 min. Furthermore, the total polyphenol content of the ozonated pineapple and bananas was higher by 9.5% and 23.5%, respectively, compared to non-treated fruit. The process of ozonation under properly selected conditions may influence an increase in ascorbic acid content in the plant material [21,27]. Ozone treatment on the buckthorn berries resulted in changes in ascorbic acid content after 24 h of the conducted process (Figure 2C). However, this impact was different depending on the concentration of the ozone used and the duration of the process. The best results were obtained for 30 min using ozone at a concentration of 10 ppm. At that time, the ascorbic acid content in the tested material was higher by 12.7% compared to the control sample (non-ozonated fruit). However, when the ozone concentration was increased to 100 ppm (30 min), a significant decrease in the ascorbic acid content was observed. It is likely that the observed effects of the oxidation of ascorbic acid was the result of the activation of ascorbate oxidase being maintained by stress related to the action of ozone gas. This enzyme degrades ascorbic acid to dehydroascorbic acid [28]. The degradation of ascorbic acid under the influence of inappropriate selection of process conditions for the type of ozonated plant material was also confirmed by others. Alothman et al. [26], treating guava fruit with ozone with a flow rate of 8 ± 0.2 mL s^−1^ for 30 min, noted a decrease in ascorbic acid by 67.1% compared to fruit not exposed to this gas (control test). The increase in the content of ascorbic acid in the plant material subjected to the ozone treatment in properly selected conditions (concentration and time of ozone exposure) was also confirmed in other studies. Piechowiak et al. [15], while ozonating raspberry fruits with ozone at a concentration of 8–10 ppm for 30 min, observed an increase in ascorbic acid content in fruits subjected to the ozonation process. The performed statistical analysis showed a significant influence of the interaction of ozone concentration and ozonation time on the ascorbic acid content in sea buckthorn berries (LSD_0.05_ = 12.46).

### 2.4. Microbial Load in the Raw Material

The ozone treatment resulted in a reduction of the microbial load in the plant material after 24 h of the conducted process (Table 2). The results of microbiological and statistical analyses showed the impact of ozone on the total number of aerobic bacteria colonies, in addition to yeasts and molds. Exposure of sea buckthorn berries to the effect of gaseous ozone at a concentration of 100 ppm for 30 min resulted in the lowest number of colonies of aerobic bacteria (cfu g^−1^). The applied process conditions resulted in the reduction of the number of aerobic bacteria colonies by 3 log cfu g^−1^ compared to the control (non-ozonated) sample. Similar effects of gaseous ozone were observed in the case of reducing the number of yeast and mold colonies on the surface of ozonated sea buckthorn berries. The number of yeast and mold colonies after the application of 100 ppm gaseous ozone for 30 min decreased by 1 log cfu g^−1^ compared to non-ozone fruit. The reduction of the microbial load was due to the antimicrobial effect of gaseous ozone, which results from its strong oxidizing properties [24]. The influence of the ozonation process on the reduction of the microbial load of fruits has been confirmed in the studies of other authors. Piechowiak et al. [19] found a decrease in the total number of mesophilic aerobic bacteria, by 1.18 log cfu g^−1^, at 48 h after the first ozone treatment of raspberry fruit (8–10 ppm for 30 min every 12 h), compared to the control.

### 2.5. Profile of Volatile Compounds

**Figure 3:**
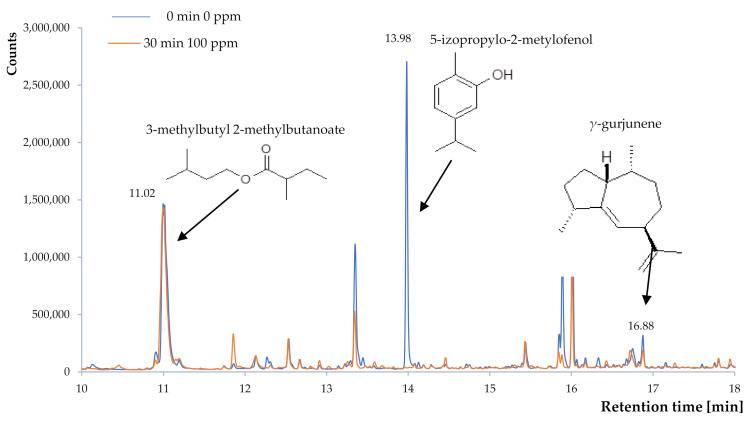
Chromatogram SPME-GC for the volatile fraction of *Hippophae rhamnoides* L. fruit.

The ozonation process affected the chemical composition of the sea buckthorn berries. The group of compounds that are particularly susceptible to decomposition under the influence of this process are volatile compounds (Table 3). In sea buckthorn fruits, these compounds belong to main two groups: organic acid esters (compounds with a fruity smell) and terpene derivatives. The first group of compounds are aliphatic derivatives that are resistant to the oxidation process. HS-SPME analysis showed that the percentages of these substances vary depending on the ozonation conditions used, but their mutual proportions are similar. The differences in the percentage composition are due to the loss of other compounds that were most likely susceptible to the ozone decomposition process. The greatest differences were observed for carvacrol presence. Carvacrol is a phenol derivative that was identified only in the control sample (non-ozonated fruit). This is an interesting observation because the aromatic compounds show a moderate tendency to ozonolysis [29]. The process carried out with the use of gaseous ozone mainly affects the surface of the ozonated raw material. The secondary metabolites are often found on the surface of the fruit in tissues capable of storing hydrophobic substances. They are often components of the cuticle, which is a barrier rich in hydrophobic compounds. Terpenes are hydrophobic substances that are often found in the cuticle. One of the components of the volatile fraction of sea buckthorn berries is carvacrol. Carvacrol is a component with a low boiling point and a relatively high vapor pressure, which makes the compound susceptible to the surface action of ozone. The HS-SPME technique used makes it possible to identify volatile compounds present in the headland phase. The lack of carvacrol in this phase in the case of ozonated fruit (Figure 3) indicates that it has been removed from the top layers of sea buckthorn berries, but this does not exclude its presence in deeper structures. It should be noted, however, that the lack of this substance in the headspace will likely affect the subjective smell of ozonated fruits. A cursory sensory analysis did not reveal the absence of foreign odors. Ozonolysis can lead to the formation of carbonyl derivatives, which often exhibit strong aromatizing properties. These compounds can arise as oxidation products of unsaturated fatty acids in the deeper layers of plant tissues [30,31]. However, they arise from long-chain unsaturated hydrocarbon derivatives. In the case of sea buckthorn berries, the aromatic hydrocarbon derivative (carvacrol) was degraded, and presumably degraded into highly volatile compounds (glyoxal), the presence of which was not detected. No significant losses of the esters, which are mainly responsible for the fruity fragrance, were observed during the ozonation process. As shown by Slynko et al. [32], these esters are the main ingredients in the scent of sea buckthorn berries and represent 71.45% of the ingredients of Hippophae scents. In the case of ozonated fruit, the proportion of these esters ranges from 97% (100 ppm, 30 min) to 60.98% for non-ozonated fruit.

## 3. Materials and Methods

### 3.1. Plant Material

The research material was acquired from a local producer in a neighborhood of Rzeszów (southern Poland) after harvest. The plant material was analyzed at the Department of Food Chemistry and Toxicology University of Rzeszów.

### 3.2. Ozone Exposure

Apparatus for the ozone treatment of plant material was fed with ozone generated by a TS 30 (Ozone Solution, Hull, MA, USA) ozone generator, and the ozone concentration was measured by a 106 M UV Ozone Solution detector (Ozone Solution, Hull, MA, USA), with the range of 0–1000 ppm [33]. The plant material was placed into the reactor chamber. Samples (500 g) of the sea buckthorn were exposed to ozone at the concentration of 10 ppm and 100 ppm for 10, 15, and 30 min respectively with the gas flow rate of 4 m^3^ h^−1^ at room temperature (20 °C). The experiments were conducted in triplicate.

### 3.3. Determination of the Moisture Content

The moisture content in the plant material was determined using a SLW 115 SMART moisture analyzer (POL-EKO-APARATURA, Wodzisław Śląski Poland). The maximal temperature during this procedure was 105 °C.

### 3.4. Measurement of Mechanical Properties

*Hippophae rhamnoides* L. samples were subjected to the puncture strength test with a pen puncture probe with a diameter of: a~8 mm, b~6 mm, using Zwick/ Roell Z010 machine (Zwick Roell Polska Sp. z o.o. Sp. K., Wrocław, Poland). Measurements of the mechanical properties were conducted at the preliminary power F = 1 N (initial force), and the speed of traverse of the beam load cell V = 0.3 mm∙mins^−1^ (crosshead return speed). The measurements were conducted in 36 repetitions for each of the analyzed varieties, on the fresh and the ozonated fruit after the first, the fourth, and the seventh day of storage at a temperature of 4 °C.

### 3.5. Content of Bioactive Compounds

The content of polyphenols in *Hippophae rhamnoides* L. fruit was measured in accordance with the methodology described by Matłok et al. [34] utilizing the Folin–Ciocalteu method. The total ascorbic acid content and the antioxidant activity DPPH were determined according to the methodology described by Oszmiański et al. [35] and Piechowiak et al. [19]. The analysis was performed in three replicates.

### 3.6. Microbiological Analysis

Microbiological analyses consisting of the indication of the total number of yeasts (cfu g^−1^) and mold and aerobic bacteria (cfu g^−1^) were carried out in accordance with the methods described by Matlok et al. [36]. The analyses were performed in three replications.

### 3.7. Head Space-Solid Phase Microextraction (HS-SPME) and Chromatographic Analysis

Head Space–Solid Phase Microextraction (HS-SPME) and chromatographic analysis was conducted in accordance with the method described by Matłok et al. [36]. The analyses were performed in three replications.

### 3.8. Statistical Analysis

The significance of the differences was checked for each term of analysis. The results were statistically processed using the analysis of variance in a two-factor random block design. Furthermore, the effect of storage time on the specified quality parameter of the control and ozonated fruit in terms of the value of the specified mechanical quality parameter and moisture content was determined using the analysis of variance in a three-factor random blocks design. The significance of differences with LSD of 0.05 was evaluated using Tukey’s multiple test. Statistical analysis of results was carried out using the ANALWAR—5.3 FR programme by Franciszek Rudnicki (University of Life Science and Technology in Bydgoszcz, Poland)

## 4. Conclusions

The results of the present study show the effect of ozone treatment on the quality of sea buckthorn (*Hippophae rhamnoides* L.) The study shows that the application of gaseous ozone leads to reduced microbial load and loss of water, and improved mechanical properties in the plant material. Furthermore, the findings show increased ascorbic acid content after ozone treatment (for 10 ppm at 30 min and 100 ppm at 15 min), and higher total content of polyphenols. Moreover, the observed increase was dependent on the applied dose of ozone. The current findings show that ozone treatment may effectively be used to extend the shelf life of sea buckthorn.

## Figures and Tables

**Table 1:** Effect of ozone treatment concentration (OC), ozone exposure time (OE), and storage time (S) on the mechanical parameters of *Hippophae rhamnoides* L. fruit.

Parameter	Treat		Storage [Days]		
	Ozone Concentration [ppm]	Ozone Exposure [min]	1	4	7
F_max_ [N]	0	0	6.82	6.20	6.11
	10	5	6.06 ^de^	6.26 ^de^	5.86 ^e^
		15	7.31 ^bcde^	8.05 ^abc^	8.17 ^ab^
		30	7.45 ^bcde^	6.99 ^cde^	8.30 ^a^
	100	5	6.46 ^cde^	6.28 ^e^	6.57 ^cde^
		15	7.69 ^bcd^	7.36 ^abcde^	7.71 ^bd^
		30	7.53 ^bc^	8.45 ^b^	9.69 ^a^
	LSD_0.05_ for: OC = 0.13; OE = 0.23; S = 0.17; OC/OE = 0.30; OC/S = n.s; * OE/S = 0.30				
dl to F_max_ [mm]	0	0	2.32	2.30	2.06
	10	5	2.65 ^abc^	2.37 ^abcd^	2.44 ^ab^
		15	2.30 ^abcd^	2.34 ^cd^	2.02 ^d^
		30	2.80 ^ab^	2.41 ^abcd^	2.60 ^abc^
	100	5	2.57 ^abc^	2.58 ^abc^	2.26 ^bcd^
		15	2.82 ^ab^	2.59 ^abc^	2.42 ^abc^
		30	2.69 ^abc^	2.87 ^a^	2.45 ^abc^
	LSD_0.05_ for: OC = 0.02; OE = 0.04; S = 0.05; OC/OE = 0.05,OC/S = 0.06; OE/S = n.s				
W to F_max_ [J]	0	0	5.22	4.67	4.32
	10	5	6.48 ^abc^	6.51 ^abc^	6.22 ^abc^
		15	4.80 ^bcd^	4.48 ^cd^	3.91 ^d^
		30	7.29 ^a^	6.17 ^abc^	7.05 ^a^
	100	5	5.76 ^abcd^	5.39 ^abcd^	5.63 ^abcd^
		15	6.88 ^ab^	6.21 ^abc^	6.09 ^abc^
		30	6.78 ^abc^	6.60 ^a^	6.61 ^abc^
	LSD_0.05_ for: OC = 0.10; OE = 0.24; S = 0.24; OC/OE = 0.29; OC/S = n.s; OE/S = 0.41				
F_max_/L_max_ [N/mm^2^]	0	0	11.93	16.65	15.29
	10	5	11.30 ^i^	26.06 ^a^^b^	18.75 ^def^
		15	13.23 ^ghi^	30.07 ^a^	18.63 ^defg^
		30	9.65 ^i^	18.48 ^defg^	19.86 ^cdeg^
	100	5	11.97 ^i^	12.20 ^hi^	13.17 ^fghi^
		15	11.97 ^hi^	17.17 ^efgh^	23.72 ^bc^
		30	14.38 ^efghi^	23.09 ^bc^	19.50 ^def^
	LSD_0.05_ for: OC = 0.55; OE = 0.62; S = 0.68; OC/OE = 0.90; OC/S = 0.96; OE/S = 1.15				

Note: * n.s.—non-significant difference; Fmax—destructive force (N), dL to Fmax—deflection at the moment of destruction (mm), W to Fmax—energy required for destruction (J), Fmax/Lmax—destructive force measurement (N/mm^2^). ^a,b,c,d,e,f,g,h,i^—statistically significant differences for the effect: ozone concentration (ppm) × ozone exposure (min) × storage (days).

**Table 2:** Microbiological load of sea buckthorn berries 24 h after the ozonation process (*n* = 3).

Parameter	Treat		Storage
	Ozone Concentration [ppm]	Ozone Exposure [min]	1 Day
**Count of yeast and mould [cfu g^−1^]**	0	0	4.82
	10	5	4.07 ^c^
		15	3.91 ^d^
		30	4.26 ^a^
	100	5	4.14 ^b^
		15	3.55 ^e^
		30	3.55 ^e^
	LSD_0.05_: OC = 2.64; OE =2.98; OC/OE= 2.92		
**Count of aerobic bacteria [cfu g^−1^]**	0	0	6.28
	10	5	6.71 ^a^
		15	5.10 ^c^
		30	4.61 ^c^
	100	5	5.84 ^b^
		15	3.64 ^c^
		30	3.63 ^c^
	LSD_0.05_: OC = 4.96; OE = 5.07; OC/OE= 5.15		

Note: LSD for α = 0.05 for the impact of ozone treatment concentration (OC), ozone exposure time (OE), and the interaction between tested parameters (OC)/(OE). ^a,b,c,d,e^ statistically significant differences for the effect: ozone concentration (ppm) × ozone exposure (min).

**Table 3:** Effect of ozone treatment concentration and ozone exposure time on chemical composition of headspace fractions of *Hippophae rhamnoides* L. fruit after one day of storage (*n* = 3).

No.	RT [min]	Peak Share in the Chromatogram [%]							Ordinary Substance Name	Systematic Substance Name	No CAS
		0 ppm 0 min	10 ppm 5 min	10 ppm 15 min	10 ppm 30 min	100 ppm 5 min	100 ppm 15 min	100 ppm 30 min			
1	8.93	trace	trace	4.49 ^a^	trace	3.46 ^b^	trace	trace	ethyl caproate	ethyl hexanoate	123-66-0
1	10.91	2.05 ^b^	3.56 ^b^	3.66 ^b^	2.32 ^b^	5.30 ^a^	trace	trace	(*E*)-sabinene hydrate	(2R,5R)-2-methyl-5-propan-2-ylbicyclo (3.1.0) hexan	17699-16-0
2	10.99	trace	trace	trace	trace	trace	trace	trace	2-methylbutyl pentanoate	2-methyl butyl valerate	55590-83-5
3	11.02	32.77 ^b^	43.77 ^b^	39.84 ^b^	51.83 ^b^	47.80 ^b^	43.88 ^b^	64.02 ^a^	isoamyl 2-methyl butyrate	3-methylbutyl 2-methylbutanoate	27625-35-0
4	11.19	0.51 ^a^	trace	0.77 ^a^	trace		2.64 ^a^	trace	ipsenol	2-methyl-6-methylideneoct-7-en-4-ol	35628-05-8
5	12.13	1.06 ^b^	trace	4.72 ^a^	trace	1.73 ^b^	9.01 ^b^	trace	ethyl benzoate	ethyl benzoate	93-89-0
6	12.27	1.55 ^a^	trace	trace	trace	trace	8.58	trace	4-carvomenthenol	4-methyl-1-propan-2-ylcyclohex-3-en-1-ol	562-74-3
7	12.53	2.52 ^b^	3.57 ^b^	5.83 ^a^	2.65 ^b^	4.77 ^a^		5.24 ^a^	ethyl caprylate	ethyl octanoate	106-32-1
9	13.35	10.25 ^a^	10.89 ^a^	13.85 ^a^	13.06 ^a^	10.37 ^a^	9.66 ^a^	8.91 ^a^	isoamyl caprylate	isoamyl octanoate	2035-99-6
9	13.98	20.37	nd	nd	nd	nd	nd	nd	carvacrol	5-izopropylo-2-metylofenol	499-75-2
10	15.44	1.75 ^b^	2.44 ^b^	3.23 ^b^	3.52 ^b^	2.26 ^b^	tarce	5.42 ^a^	benzyl valerate	benzyl pentanoate	10361-39-4
11	15.85	2.18 ^b^	3.29 ^b^	2.10 ^b^	4.18 ^a^	2.76 ^b^	trace	trace	*α*-gurjunene	(2S,4R,7R,8R)-3,3,7,11-tetramethyltricyclo[6.3.0.02.4]undec-1(11)-ene	489-40-7
12	15.89	8.54 ^a^	7.69 ^a^	3.86 ^b^	5.64 ^b^	5.97 ^b^	trace	trace	caryophyllene	(1R,4E,9S)-bicyclo[7.2.0]undec-4-ene, 4,11,11-trimethyl-8-methylene-,	87-44-5
13	16.01	11.64 ^a^	15.48 ^a^	12.30 ^a^	15.79 ^a^	14.67 ^a^	16.94 ^a^	12.78 ^a^	isoamyl benzoate	3-methylbutyl benzoate	94-46-2
14	16.75	1.79 ^a^	3.58 ^a^	trace	trace	trace	trace	trace	*β*-selinene	(3S,4aR,8aS)-8a-methyl-5-methylidene-3-prop-1-en-2-yl-1,2,3,4,4a,6,7,8-octahydronaphthalen	17066-67-0
15	16.88	2.01 ^a^	2.08 ^a^	3.97 ^a^	trace	trace	7.85 ^a^	2.29 ^a^	(1R,4R,7R)-1,4-dimethyl-7-prop-1-en-2-yl-1,2,3,3a,4,5,6,7-octahydroazulene	*γ*-gurjunene	22567-17-5
TOTAL		98.99	96.35	98.62	98.99	98.60	98.56	98.66			

Note: ^a, b^—difference at significant level *p* < 0.05.
